# Shear Wave Dispersion Predicts Liver Fibrosis and Adverse Outcomes in Patients with Heart Failure

**DOI:** 10.3390/jcm9123953

**Published:** 2020-12-06

**Authors:** Himika Ohara, Akiomi Yoshihisa, Shinji Ishibashi, Mitsuko Matsuda, Yukio Yamadera, Yukiko Sugawara, Yasuhiro Ichijo, Yu Hotsuki, Koichiro Watanabe, Fumiya Anzai, Yu Sato, Yusuke Kimishima, Tetsuro Yokokawa, Tomofumi Misaka, Takamasa Sato, Masayoshi Oikawa, Atsushi Kobayashi, Yasuchika Takeishi

**Affiliations:** 1 Department of Cardiovascular Medicine, Fukushima Medical University, Fukushima 960-1295, Japan; h-oohara@fmu.ac.jp (H.O.); yuki11@fmu.ac.jp (Y.S.); ic-yasu@fmu.ac.jp (Y.I.); yhotsuki@fmu.ac.jp (Y.H.); koichi-w@fmu.ac.jp (K.W.); anzaif14@fmu.ac.jp (F.A.); yu-sato@fmu.ac.jp (Y.S.); kimishi@fmu.ac.jp (Y.K.); yokotetu@fmu.ac.jp (T.Y.); misaka83@fmu.ac.jp (T.M.); takamasa@fmu.ac.jp (T.S.); moikawa@fmu.ac.jp (M.O.); koba-a@fmu.ac.jp (A.K.); takeishi@fmu.ac.jp (Y.T.); 2 Department of Advanced Cardiac Therapeutics, Fukushima Medical University, Fukushima 960-1295, Japan; 3 Department of Clinical Laboratory Medicine, Fukushima Medical University, Fukushima 960-1295, Japan; s-bashi@fmu.ac.jp (S.I.); mda-m@fmu.ac.jp (M.M.); yamadera@fmu.ac.jp (Y.Y.)

**Keywords:** viscosity, fibrosis, prognosis, abdominal ultrasonography, liver function test, right heart catheterization

## Abstract

Background: It has been recently reported that liver stiffness assessed by transient elastography reflects right atrial pressure (RAP) and is associated with worse outcomes in patients with heart failure (HF). However, the relationship between shear wave dispersion (SWD, a novel indicator of liver viscosity) determined by abdominal ultrasonography and RAP, and the prognostic impact of SWD on HF patients have not been fully examined. We aimed to clarify the associations of SWD with parameters of liver function test (LFT) and right heart catheterization (RHC), as well as with cardiac events such as cardiac death and worsening HF, in patients with HF. Methods: We performed abdominal ultrasonography, LFT and RHC in HF patients (*n* = 195), and followed up for cardiac events. We examined associations between SWD and parameters of LFT and RHC. Results: There were significant correlations between SWD and circulating levels of direct bilirubin (R = 0.222, *p* = 0.002), alkaline phosphatase (R = 0.219, *p* = 0.002), cholinesterase (R = −0.184, *p* = 0.011), and 7S domain of collagen type IV (R = 0.177, *p* = 0.014), but not with RAP (R = 0.054, *p* = 0.567) or cardiac index (R = −0.015, *p* = 0.872). In the Kaplan–Meier analysis, cardiac event rate was significantly higher in the high SWD group (SWD ≥ 10.0 (m/s)/kHz, *n* = 103) than in the low SWD group (SWD < 10.0 (m/s)/kHz, *n* = 92; log-rank, *p* = 0.010). In the Cox proportional hazard analysis, high SWD was associated with high cardiac event rates (hazard ratio, 2.841; 95% confidence interval, 1.234–6.541, *p* = 0.014). In addition, there were no interactions between SWD and all subgroups, according to the subgroup analysis. Conclusions: SWD assessed by abdominal ultrasonography reflects liver fibrosis rather than liver congestion, and is associated with adverse prognosis in HF patients.

## 1. Introduction

The number of patients with heart failure (HF) has been rapidly increasing to an estimated 26 million patients worldwide [1,2,3,4]. HF and liver dysfunction can coexist owing to complex cardiohepatic interactions including the development of hypoxic hepatitis (reduced arterial perfusion) and congestive hepatopathy (passive congestion) in HF patients [5,6,7]. Liver congestion and fibrosis might be mutually associated with liver stiffness [5,6,7,8]. To evaluate liver stiffness using image testing non-invasively, measurement of shear-wave propagation velocity, namely, shear-wave elastography (SWE), has been recently reported [9,10,11,12]. SWE uses acoustic radiation force to create laterally propagating shear waves that can be traced to determine the SW propagation velocity [13,14,15,16]. It has been reported that liver stiffness assessed by SWE reflects right atrial pressure (RAP) and is associated with worse outcomes in patients with HF [8,17,18,19].

On the other hand, in clinical hepatology, prior to liver fibrosis, inflammation, increased biliary pressure and congestion occur [20,21]. It has been reported that SWE mainly reflects the degree of fibrosis, and that shear wave dispersion (SWD) can evaluate the degree of inflammation, necrosis and fatty deposition more accurately than SWE [22,23]. Although SWE reflects both elasticity and viscosity (i.e., viscoelasticity) [22]. SWD is calculated based on SWD measurements and reflects viscosity only [24,25]. Since liver fibrosis advances gradually as hepatitis progresses [26], SWD may be able to estimate liver damage in an earlier stage than SWE. In this regard, SWD is gathering attention in terms of superiority to SWE in patients with chronic liver disease [27].

In patients with HF, we hypothesize that SWD (viscosity) seems to reflect tissue congestion and inflammation more accurately than SWE (viscoelasticity) and could detect functional liver disorder more accurately in HF patients. However, the relationship between SWD and RAP, and the prognostic impact of liver viscosity on HF patients have not yet been examined. Therefore, we aimed to clarify the associations of SWD with parameters of liver function test (LFT) and right heart catheterization (RHC), as well as with cardiac events such as cardiac death and worsening HF, in patients with HF.

## 2. Experimental Section

### 2.1. Subjects and Study Protocol

This was a prospective observational study of 232 decompensated HF patients who had undergone abdominal ultrasonography and were discharged from Fukushima Medical University Hospital between June 2018 and March 2020. Diagnosis of decompensated HF was made by each patient’s attending cardiologist based on the established HF guidelines. Blood samples, abdominal ultrasonography and echocardiography were obtained at hospital discharge. All patients underwent testing for hepatitis B surface antigen and hepatitis C antibodies, and their medical histories were checked for chronic liver disease (cirrhosis, hepatic tumors, bile duct disease, etc.) and alcohol abuse (≥30 g/day for men, ≥20 g/day for women). We subsequently excluded those patients who had the above-mentioned liver diseases (*n* = 31), and/or were undergoing dialysis (*n* = 6). Finally, 195 patients were enrolled in this study, among whom 116 had undergone RHC within 3 days of abdominal ultrasonography. The patients were divided into two groups on the basis of the median SWD: the low SWD group (SWD < 10.0 (m/s)/kHz, *n* = 92, 47%) or the high SWD group (SWD ≥ 10 (m/s)/kHz, *n* = 103, 53%).

First, we compared the clinical features as well as the results from laboratory tests and echocardiography between the two groups. In addition, we performed a correlation analysis of the interaction between SWD levels and the parameters of laboratory tests and echocardiography. Second, the patients were followed up until July 2020 for cardiac events as composites of cardiac death or unplanned re-hospitalization for HF treatment. For patients who experienced ≥2 events, only the first event was included in the analysis. Since these patients visited the hospital monthly or every other month, we were able to follow up on all patients. Status and dates of death were obtained from the patient’s medical records. Those administering the survey were blind to the analyses, and written informed consent was obtained from all study subjects. The study protocol was approved by the Ethics Committee of Fukushima Medical University, and was carried out in accordance with the principles outlined in the Declaration of Helsinki. Reporting of the study conforms to Strengthening the Reporting of Observational Studies in Epidemiology and the Enhancing the Quality and Transparency of Health Research guidelines.

Hypertension was defined as the recent use of antihypertensive drugs, a systolic blood pressure of ≥140 mmHg, or a diastolic blood pressure of ≥90 mmHg. Dyslipidemia was defined as the recent use of cholesterol-lowering drugs, a triglyceride value of ≥150 mg/dL, a low-density lipoprotein cholesterol value of ≥140 mg/dL, or a high-density lipoprotein cholesterol value of <40 mg/dL. Chronic kidney disease was defined as an estimated glomerular filtration rate of <60 mL/min per 1.73 cm^2^, and anemia was defined as hemoglobin levels of <12.0 g/dL in women and <13.0 g/dL in men [28]. Atrial fibrillation (AF) was identified via electrocardiogram performed during hospitalization or from medical records, including past history.

### 2.2. Abdominal Ultrasonography

**Figure 1:**
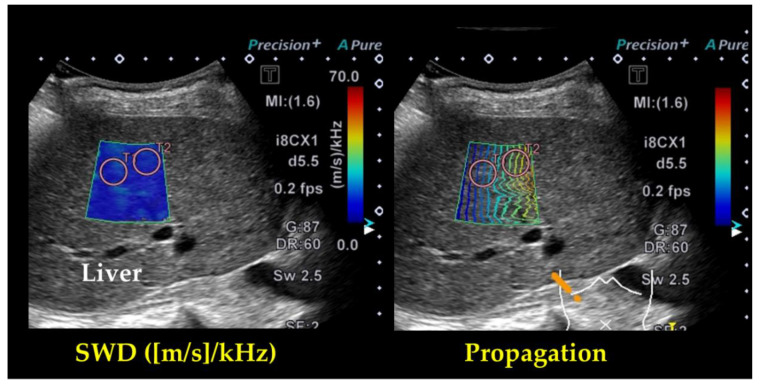
Representative images of shear wave dispersion (SWD) in a patient with heart failure (HF). A 3 × 3 cm sized sample box was placed in the liver parenchyma and then shear wave propagation was activated by using acoustic radiation force. The shear wave propagation was seen within the sample box as smooth parallel lines indicating a stable measurement condition. Two regions of interest with a diameter of 1.0 cm are placed within the sample box. The SWD on its map is as follows. T1: 11.55 (m/s)/kHz, T2: 11.27 (m/s)/kHz.

Due to the absence of guidelines in the literature for ensuring proper SWD measurements, we employed recommended 2D-SWE methods [29]. The methods of the actual acquisition are as follows. We performed abdominal ultrasonography in the patients who were in a stable condition without changes in medications at hospital discharge. Two experienced sonographers performed abdominal ultrasonography (S.I, with 15 years of experience in abdominal ultrasonography, and M.M, with 23 years of experience) who were blinded to all clinical data. The patients fasted for at least 12 h before the abdominal ultrasonography, and were placed in the supine position. The transducer was placed in the intercostal space, and the patient’s right arm was raised above the head to obtain a proper acoustic window. The tip of the probe transducer was placed perpendicular to the liver capsule to avoid refraction of the acoustic radiation force impulse. The measurement was made while the patients were holding their breath. A sample box (we usually define it as an area of 3 × 3 cm) was placed 1.5–2.0 cm below the liver capsule to avoid reverberation artifacts and increased subcapsular stiffness. Within the sample box, two regions of interest for the measurement (we usually use a region of interest with a diameter of 1.0 cm) were placed manually to avoid large blood vessels, bile ducts and masses within the reference propagation map (Figure 1). Measurements were performed seven times, and the median value was defined as the SWD ((m/s)/kHz).

### 2.3. Echocardiography

Echocardiography was performed by experienced echocardiographers using standard techniques [30]. The echocardiographic parameters included left ventricular ejection fraction (LVEF), left atrial volume, early transmitral flow velocity to mitral annular velocity ratio (mitral valve E/e’), right atrium and ventricle areas, right ventricular fractional area change (RV-FAC), inferior vena cava diameter, tricuspid regurgitation pressure gradient (TRPG), tissue Doppler-derived tricuspid lateral annular systolic velocity (tricuspid valve S’), and tricuspid annular plane systolic excursion (TAPSE) [31]. The LVEF was calculated using Simpson’s method. The RV-FAC, defined as (end-diastolic area—end-systolic area)/end-diastolic area × 100, was a measure of right ventricular systolic function [31]. All measurements were performed using ultrasound systems (ACUSON Sequoia, Siemens Medical Solutions USA, Inc., Mountain View, CA, USA).

### 2.4. Right Heart Catheterizations and Hemodynamic Measurements

Of the total 195 patients, RHC was partly performed based on the remedial judgment of the attending physician in 116 patients. The RHCs were all performed within 3 days of abdominal ultrasonography, and with the patients in a stable condition without changes in medications, including doses, similar to the setting for abdominal ultrasonography. All RHCs were performed with the patients, in a resting supine position under fluoroscopic guidance, at room air, and at rest for more than 30 min after catheter placement. Mean RAP and cardiac output were measured using a 7F Swan–Ganz catheter (Edwards Lifesciences, Irvine, CA, USA). Cardiac output was calculated based on the direct Fick method [32].

### 2.5. Measurement of Laboratory Data

The B-type natriuretic peptide (BNP) levels were measured using a specific immunoradiometric assay (Shionoria BNP kit, Shionogi, Osaka, Japan). The serum 7S domain of collagen type IV (P4NP 7S), which indicates liver fibrosis, was measured by radioimmunoassay (Type IV collagen 7S kit, SCETI MEDICAL LABO K.K., Tokyo, Japan) [32,33]. These assays were blindly performed by experienced laboratory technicians.

### 2.6. Statistical Analysis

Normally distributed data are presented as mean ± standard deviation, and nonnormally distributed data are presented as median and interquartile range. The categorical variables are expressed as numbers and percentages, and the Chi-square test was used for their comparisons. Parametric variables were compared using Student’s *t*-test, and nonparametric variables were compared using the Mann–Whitney U test. Correlations between SWD and the parameters of laboratory data, echocardiography or RHC, were assessed using Pearson’s correlation analysis for parametric variables and Spearman’s correlation analysis for non-parametric variables. Kaplan–Meier analysis was used with a log-rank test to assess cardiac event rates as composites of cardiac death or unplanned re-hospitalization for HF treatment. These curves helped in identifying non-proportionality patterns in hazard function such as convergence (difference in risk between the groups decreases with time), divergence, or crossing of the curves. In addition, a Schoenfeld test for the violation of proportional hazards, which can be used to assess the correlation between scaled residuals and time, was also conducted. We assessed SWD as a predictor for post-discharge cardiac events using the Cox proportional hazard analysis. To adjust clinical confounding factors, because of the small number of events and sample size, as well as the presence of multicollinearity, we did not perform multivariate Cox proportional hazard analysis, but instead performed subgroup analysis. The univariate Cox proportional hazard analysis led to subdivision into subgroups based on the presence or absence of categorical factors, and the median of continuous variables. Interaction *p* values were obtained using a multivariate model including SWD, subgroup factors, and interactions between SWD and subgroup factors. In the subgroup analysis, we considered *p* values of < 0.0028, which were determined by the Bonferroni correction, as statistically significant for all comparisons. In all comparisons other than those in the subgroup analysis, *p* values < 0.05 were considered statistically significant. These analyses were performed using a statistical software package (SPSS ver. 24.0, IBM, Armonk, NY, USA).

## 3. Results

**Figure 2:**
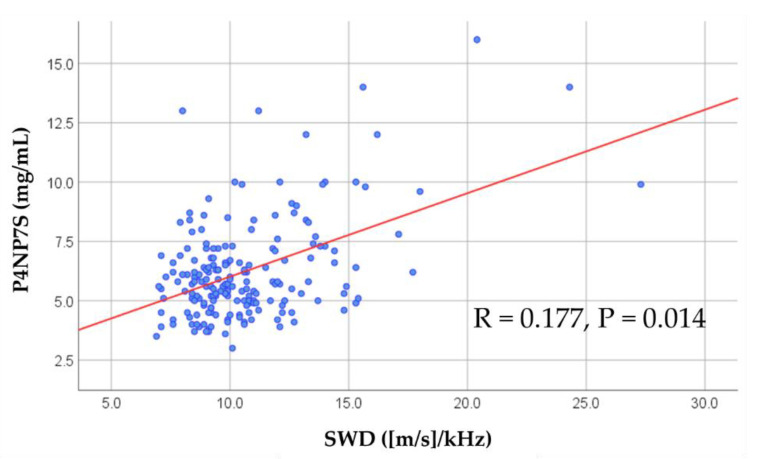
Correlation between SWD and P4NP 7S. SWD indicates shear wave dispersion and P4NP 7S indicates 7S domain of collagen type IV.

Comparisons of patient characteristics between the low and high SWD groups are summarized in Table 1. The patients in the high SWD group were older, had a lower body mass index, and a higher prevalence of AF. In contrast, sex, blood pressure, heart rate, NYHA class, and other comorbidities, including coronary artery disease, hypertension, diabetes mellitus and anemia, did not differ between the groups. With regard to laboratory data, levels of direct bilirubin, alkaline phosphatase (ALP) and P4NP 7S were higher, and cholinesterase levels were lower in the high SWD group than in the low SWD group. However, total protein, albumin, aspartate aminotransferase, alanine aminotransferase, C-reactive protein, BNP, creatinine, estimated glomerular filtration rate and sodium did not differ between the two groups. Regarding the parameters of echocardiography and RHC, left atrial volume, mitral valve E/e′, right atrial end-systolic area, and inferior vena cava diameter were higher in the high SWD group than in the low SWD group. In contrast, LVEF, RV-FAC, TRPG, tricuspid valve S’, TAPSE, RAP, mean pulmonary artery pressure, and cardiac index were similar between the groups. In addition, there were significant associations between SWD and age, body mass index, the prevalence of AF and chronic kidney disease, levels of direct bilirubin, ALP, cholinesterase, P4NP 7S, and left atrial volume, but not with RAP or cardiac index. As shown in Figure 2, SWD was significantly correlated with P4NP 7S (R = 0.177, *p* = 0.014). These results suggest that elevated SWD indicates liver fibrosis, rather than liver congestion.

**Figure 3:**
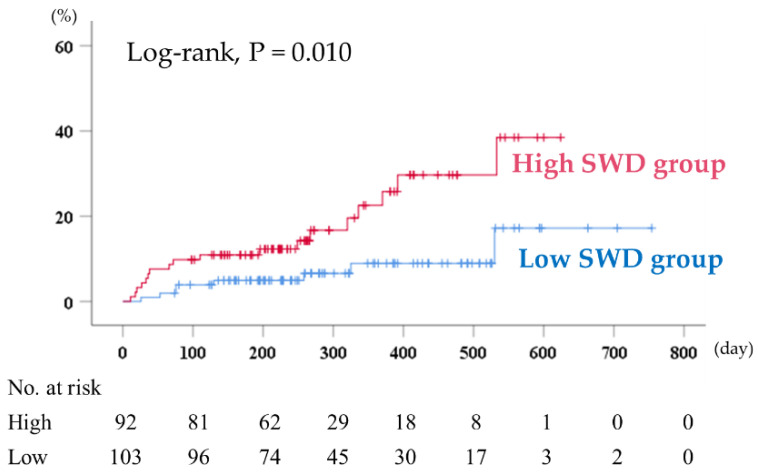
Kaplan–Meier analysis for cardiac event rates stratified by SWD levels. SWD indicates shear wave dispersion.

During the follow-up period (median 259 days; range 11–754 days), 26 cardiac events, including 5 cardiac deaths and 23 unplanned hospitalizations due to HF, occurred. As shown in Figure 3, in the Kaplan–Meier analysis, cardiac event rates were significantly higher in the high SWD group than in the low SWD group (log-rank, *p* = 0.010). In the univariate Cox proportional hazard analysis, high SWD was associated with high cardiac event rates (hazard ratio, 2.841; 95% confidence interval, 1.234–6.541, *p* = 0.014). In addition, there were no interactions between SWD and all subgroups (e.g., HFrEF, HFmrEF and HFpEF), according to the subgroup analysis (Table 2).

## 4. Discussion

In this study, we investigated the associations of SWD assessed by abdominal ultrasonography with LFT, RHC and echocardiography, as well as its prognostic impact in HF patients. The present study is, to the best of our knowledge, the first to report that the SWD, a marker of liver viscosity, is associated with higher cardiac events in HF patients.

Cardio-hepatic interaction has been reported. Increased central venous pressure causes hepatocyte atrophy and perisinusoidal edema in the liver, namely, hepatic congestion [5,7,34,35]. In HF patients, increased pressure within the hepatic sinusoid favors bile duct damage by disrupting endothelial cells, and the inter hepatocyte tight junctions that separate the extravascular space from the bile canaliculus. Further, stagnant flow favors thrombosis within sinusoids, hepatic venules, and portal tracts, thereby contributing to liver fibrosis [6,36]. Centrilobular necrosis can extend to peripheral areas and cause deposition and spread of connective tissue, bridging one central vein to another, finally leading to liver cirrhosis [6]. Cardiogenic ischemic hepatitis, known also as acute cardiogenic liver injury, is a clinical and histological syndrome leading to the reduction of hepatic blood flow due to acute fall in cardiac output [34,35]. Reduced hepatic blood flow can cause hypoxic hepatopathy, and hypoxia can cause centrilobular necrosis and sinusoidal damage in the liver, and leads to the elevation of transaminase [35].

With regards to LFT, liver dysfunction, such as the elevation of serum bilirubin, transaminase, ALP, gamma-glutamyl transferase and P4NP 7S, frequently occurs in right-sided HF, and is associated with disease severity and prognosis [6,37,38,39,40]. On the other hand, liver image testing (i.e., SWD) might reflect liver damage and histological findings and may predict prognosis more accurately than LFT.

In the present study, contrary to our expectations, high SWD was associated with liver fibrosis (P4NP 7S), but not with increased central venous pressure (i.e., RAP, inferior vena cava, TRPG), inflammation, necrosis (i.e., transaminase, gamma-glutamyl transferase) and hypoperfusion (i.e., cardiac index). Possible explanations are as follows: (1) Since we performed abdominal ultrasonography, echocardiography and RHC in HF patients who were in a stable condition, we may have failed to detect the peak of the increased central venous pressure in acute decompensated HF, and SWD might have reflected remaining fibrosis after improvement of congestion. (2) In general, liver fibrosis increases gradually in parallel with the stage progression of chronic liver disease. However, shear waves propagate disproportionally as fibrosis progresses [22,25,41,42,43]. Namely, SWD is increased in hepatitis patients, whereas SWD is decreased in cirrhosis patients (with grade 4 or 5 severe fibrosis) [41,44]. Thus, SWD is not an indicator of the degree of liver fibrosis in subjects with severe fibrosis. Fortunately, since we excluded distinct liver disease (including severe fibrosis) in the present study, we were able to precisely measure SWD, and observed associations between SWD and P4NP 7S (liver fibrosis). It has been reported that SWD decreases with the improvement of hepatic lobule inflammation by risk factor management in patients with nonalcoholic fatty liver disease [25]. HF patients with high SWD might indicate liver fibrosis, and decreasing central venous pressure by diuretic agents or anti-inflammatory therapy by statins, etc., might be useful to improve prognosis in such patients

## 5. Study Limitations

The current study has several limitations. First, as a prospective cohort study of a single center with a relatively small number of patients and a short follow-up period, the study may be somewhat underpowered. Although we performed subgroup analysis, we could not fully adjust the confounding factors. Second, although HF patients with documented liver disease were excluded, we were unable to completely exclude the presence of liver diseases, which may have affected the hepatic ultrasound imaging results. Therefore, if these HF patients already had disorders of liver stiffness or fibrosis, it would not be related to only increased central venous pressure. The relationships between SWD and other fibrosis evaluations such as liver biopsy, which is not generally performed in HF patients, or imaging (e.g., computed tomography, magnetic resonance imaging), or biomarker other than P4NP 7S (e.g., circulating pro-collagen type I or propeptide of type III collagen) should be assessed in further studies. Third, we conducted the present study using only variables during hospitalization, without taking into consideration changes in medical parameters or treatments after discharge. Fourth, since the attending physicians were the ones who decided whether RHC should be performed, there might be a potential selection bias. Therefore, the present results should be viewed as preliminary, and further studies with a larger population are needed. Fifth, the hemodynamics of HF patients changes dynamically. Thus, we should assess alterations of SWD in response to hemodynamic changes in further studies.

## 6. Conclusions

SWD assessed by abdominal ultrasonography reflects liver fibrosis rather than liver congestion, and is associated with adverse prognosis in HF patients.

## Figures and Tables

**Table 1:** Comparisons of patient characteristics (*n* = 195).

	SWD Group (Low vs. High)			Correlation with SWD	
	Low SWD (SWD < 10.0, *n* = 103)	High SWD (10.0 ≤ SWD, *n* = 92)	*p*-Value	Correlation Coefficient	*p*-Value
**SWD ((m/s)/kHz)**	9.0 (8.4–9.4)	12.0 (10.7–13.7)	<0.001	-	-
**Demographics**					
Age (years)	70.0 (61.0–80.0)	75.5 (67.0–83.0)	0.003	0.173	0.015
Male sex (*n*, %)	38 (41.3)	54 (58.7)	0.543	0.055	0.442
Body mass index (kg/m^2^)	23.8 ± 3.3	22.2 ± 4.1	0.003	−0.208	0.003
Systolic BP (mmHg)	119.0 (108.0–137.0)	122.0 (101.0–132.0)	0.236	−0.075	0.298
Diastolic BP (mmHg)	69.0 ± 12.7	67.2 ± 12.3	0.339	−0.069	0.340
Heart rate (bpm)	67.0 (59.0–80.0)	70.0 (62.0–82.0)	0.257	0.091	0.205
NYHA class III or IV	8 (7.8)	6 (6.6)	0.753	−0.010	0.885
Past hospitalization due to HF	35 (34.0)	24 (26.1)	0.231	−0.086	0.233
The use of more than once class of a diuretic	29 (28.2)	32 (34.8)	0.319	0.071	0.322
Etiology Ischemic/myopathy/ valvular/arrhythmia/pulmonary/congenital/others	19(18.4)/28 (27.2)/ 35 (34.0)/10 (9.7)/ 7 (6.8)/ 2 (1.9)/2(1.9)	17(18.5)/16 (17.4)/45 (48.9)/8 (8.7)/3 (3.3)/ 2(2.2)/1(1.1)	0.402	-	-
**Comorbidities**					
CAD (*n*, %)	27 (26.2)	24 (26.1)	0.984	−0.032	0.653
Atrial fibrillation (*n*, %)	27 (26.2)	42 (45.7)	0.005	0.164	0.022
Hypertension (*n*, %)	70 (68.0)	59 (64.1)	0.573	0.019	0.791
Dyslipidemia (*n*, %)	75 (72.8)	57 (62.0)	0.106	−0.073	0.309
Diabetes mellitus (*n*, %)	37 (35.9)	39 (42.4)	0.355	0.071	0.323
CKD (*n*, %)	58 (56.3)	64 (69.6)	0.056	0.193	0.007
Anemia (*n*, %)	43 (41.7)	47 (51.1)	0.192	0.067	0.355
**Laboratory data**					
Total protein (g/dL)	7.0 ± 0.7	6.9 ± 0.8	0.291	−0.079	0.273
Albumin (g/dL)	4.0 (3.6–4.3)	3.8 (3.4–4.2)	0.100	−0.118	0.100
Total bilirubin (mg/dL)	0.7 (0.6–1.0)	0.8 (0.6–1.1)	0.181	0.096	0.182
Direct bilirubin (mg/dL)	0.1 (0.1–0.1)	0.1 (0.1–0.1)	0.002	0.222	0.002
AST (U/L)	21.0 (17.0–27.0)	23.0 (18.0–28.0)	0.124	0.110	0.125
ALT (U/L)	19.0 (12.0–26.0)	16.5 (13.0–23.0)	0.496	−0.049	0.498
Alkaline phosphatase (U/L)	223.0 (176.0–269.0)	257.0 (203.0–314.3)	0.002	0.219	0.002
Gamma-glutamyl transferase (U/L)	33.0 (18.0–58.0)	42.5 (21.0–82.8)	0.142	0.105	0.143
Cholinesterase (U/L)	266.0 (217.0–331.0)	232.5 (185.8–303.0)	0.011	−0.184	0.011
Log CRP (mg/dL)	−0.82 (−1.22–−0.19)	−0.66 (−1.05–−0.09)	0.090	0.122	0.090
P4NP 7S (ng/mL)	5.6 (4.7–6.5)	6.0 (5.0–8.1)	0.015	0.177	0.014
Log BNP (pg/mL)	2.26 (1.94–2.48)	2.34 (1.90–2.69)	0.141	0.095	0.185
Creatinine (mg/dL)	0.99 (0.86–1.19)	1.09 (0.82–1.34)	0.505	0.048	0.507
eGFR (mL/min/1.73 cm^2^)	52.0 (41.0–64.0)	48.5 (36.8–60.3)	0.435	−0.056	0.437
Sodium (mEq/L)	140.0 (138.0–141.0)	140.0 (139.0–141.0)	0.742	−0.024	0.743
**Echocardiography**					
LV ejection fraction (%)	55.1 ± 15.8	54.4 ± 15.7	0.858	0.023	0.753
Left atrial volume (mL)	77.8 ± 41.0	96.7 ± 55.5	0.001	0.197	0.007
Mitral valve E/e’	12.7 ± 8.3	14.8 ± 8.5	0.029	0.135	0.066
RA end-systolic area (cm^2^)	16.6 ± 6.6	20.0 ± 7.5	0.006	0.118	0.275
RV area diastole (cm^2^)	19.5 ± 7.8	21.7 ± 7.7	0.154	−0.204	0.299
RV area systole (cm^2^)	12.4 ± 6.6	13.8 ± 7.0	0.303	−0.151	0.442
RV-FAC (%)	38.1 ± 11.3	38.6 ± 11.7	0.808	−0.154	0.112
IVC (mm)	14.7 ± 3.6	16.1 ± 4.8	0.004	0.104	0.148
TRPG (mmHg)	25.3 ± 12.2	26.8 ± 12.8	0.350	0.138	0.067
Tricuspid valve S’ (cm)	10.9 ± 3.6	10.2 ± 3.3	0.161	−0.165	0.224
TAPSE	18.7 ± 4.3	18.4 ± 4.9	0.607	−0.030	0.688
**Right heart catheterization**					
RAP (mmHg)	7.0 (5.0–9.0)	7.0 (5.0–10.0)	0.564	0.054	0.567
Mean PAP (mmHg)	22.5 (17.0–33.0)	22.5 (18.0–29.0)	0.871	−0.015	0.872
Cardiac index (L/min/m^2^)	2.4 (2.1–2.9)	2.4 (2.1–2.8)	0.879	−0.015	0.880

SWD, shear wave dispersion; BP, blood pressure; NYHA, New York Heart Association; CAD, coronary artery disease; CKD, chronic kidney disease; AST, aspartate aminotransferase; ALT, alanine aminotransferase; log CRP, log-transformed C-reactive protein; Gamma-glutamyl transferase P4NP 7S, 7S domain of collagen type IV; log BNP, log-transformed B-type natriuretic peptide; eGFR, estimated glomerular filtration rate; LV, left ventricle; RA, right atrial; RV, right ventricle; RV-FAC, right ventricle fractional area change; IVC, inferior vena cava diameter; TRPG, tricuspid regurgitation pressure gradient; TAPSE, tricuspid annular plane systolic excursion; RAP, right atrium pressure; PAP, pulmonary artery pressure.

**Table 2:** Subgroup analysis for predicting cardiac events (26 events/*n* = 195): the impact of high SWD (vs. low SWD).

Factor	Subgroup	*n*	HR	95% CI	*p*-Value	Interaction *p*-Value
**Total**		**195**	**2.841**	**1.234–6.541**	**0.014**	**-**
**Demographic data**						
Age	<72	89	2.356	0.713–7.782	0.160	0.656
	≥72	106	3.466	0.976–12.309	0.055	
Sex	Male	110	1.822	0.662–5.019	0.246	0.200
	Female	85	5.679	1.203–26.815	0.028	
Body mass index	<23	96	2.079	0.738–5.853	0.166	0.269
	≥23	99	3.197	0.760–13.452	0.113	
Systolic blood pressure	<120	95	3.747	1.334–10.526	0.012	0.432
	≥120	100	1.761	0.420–7.379	0.439	
Diastolic blood pressure	<67	90	2.419	0.838–6.982	0.102	0.793
	≥67	105	3.233	0.835–12.516	0.089	
Heart rate	<69	91	1.309	0.440–3.897	0.628	0.053
	≥69	104	12.815	1.663–98.761	0.014	
NYHA functional class	I or II	180	2.555	1.041–6.272	0.041	0.598
	III or IV	14	4.546	0.349–59.192	0.248	
Past hospitalization due of HF	Yes	59	3.454	1.038–11.495	0.043	0.815
	No	136	2.732	0.856–8.719	0.090	
The use of more than once class of a diuretic	Yes	61	2.771	0.750–10.238	0.126	0.954
	No	134	2.539	0.848–7.601	0.096	
CAD	Yes	51	0.875	0.212–3.621	0.854	0.057
	No	144	4.985	1.631–15.238	0.005	
Atrial fibrillation	Yes	69	3.296	0.707–15.370	0.129	0.862
	No	126	2.687	0.948–7.612	0.063	
Hypertension	Yes	129	6.379	1.831–22.221	0.004	0.033
	No	66	0.754	0.217–3.027	0.810	
Dyslipidemia	Yes	131	4.733	1.522–14.717	0.007	0.111
	No	63	1.152	0.321–4.126	0.828	
Diabetes mellitus	Yes	75	2.723	0.954–7.773	0.061	0.993
	No	119	2.721	0.680–10.891	0.157	
CKD	Yes	122	2.830	1.026–7.808	0.045	0.760
	No	73	2.015	0.403–10.082	0.394	
Anemia	Yes	90	4.194	1.194–14.725	0.025	0.294
	No	105	1.637	0.470–5.709	0.439	
BNP	<195.4	97	2.773	0.506–15.187	0.240	0.885
	≥195.4	98	2.449	0.941–6.376	0.067	
EF	HFrEF	110	5.018	1.084–23.229	0.039	0.207
	HFmrEF	24	-	-	-	
	HFpEF	61	1.876	0.666–5.288	0.234	

HR, hazard ratio; CI, confidence interval; HFrEF, heart failure with reduced ejection fraction; HFmrEF, heart failure with mid-range ejection fraction; HFpEF, heart failure with preserved ejection fraction.
